# Acoustothermal heating of polydimethylsiloxane microfluidic system

**DOI:** 10.1038/srep11851

**Published:** 2015-07-03

**Authors:** Byung Hang Ha, Kang Soo Lee, Ghulam Destgeer, Jinsoo Park, Jin Seung Choung, Jin Ho Jung, Jennifer Hyunjong Shin, Hyung Jin Sung

**Affiliations:** 1 Department of Mechanical Engineering, KAIST, 291 Daehak-ro, Yuseong-gu, Daejeon 305-701, Korea

## Abstract

We report an observation of rapid (exceeding 2,000 K/s) heating of polydimethylsiloxane (PDMS), one of the most popular microchannel materials, under cyclic loadings at high (~MHz) frequencies. A microheater was developed based on the finding. The heating mechanism utilized vibration damping in PDMS induced by sound waves that were generated and precisely controlled using a conventional surface acoustic wave (SAW) microfluidic system. The refraction of SAW into the PDMS microchip, called the leaky SAW, takes a form of bulk wave and rapidly heats the microchannels in a volumetric manner. The penetration depths were measured to range from 210 μm to 1290 μm, enough to cover most sizes of microchannels. The energy conversion efficiency was SAW frequency-dependent and measured to be the highest at around 30 MHz. Independent actuation of each interdigital transducer (IDT) enabled independent manipulation of SAWs, permitting spatiotemporal control of temperature on the microchip. All the advantages of this microheater facilitated a two-step continuous flow polymerase chain reaction (CFPCR) to achieve the billion-fold amplification of a 134 bp DNA amplicon in less than 3 min.

The popularity of surface acoustic waves (SAWs) for microfluidic actuation and manipulation stems from its highly efficient fluid-structural/structure-structural coupling owing to the concentrated energy at the medium surface^1^. Surface vibration of a piezoelectric material through the transduction of electric fields is a common way to generate SAWs. SAWs propagating on the piezoelectric substrate readily refract into a microchannel and manipulate liquids and suspended particles, carrying out a host of microfluidic operations^2^. Polydimethylsiloxane (PDMS) has been widely used as a microchannel material for SAW microfluidic system. The PDMS microchip is not only easy to fabricate through soft lithography but can also be easily coupled with a piezoelectric substrate such as lithium niobate (LiNbO_3_). However, PDMS absorbs more acoustic energy than liquid samples and the other microchannel materials such as glass or silicon, increasing the potential of thermal damage to biological samples^3^. Apparently, there have been reports that the use of PDMS in the SAW microfluidic system substantially increased the temperature of liquid samples at high input power^4^,^5^.

We believe that this may not just be a problem. A PDMS microchip can be efficiently heated by acoustic absorption, serving as a reactor and a heater simultaneously. The conventional heating methods have had difficulties in rapidly regulating the temperature of liquid samples via conduction through the walls of PDMS microchannels due to the low thermal conductivity of PDMS. On the other hand, the leaky SAWs absorbed by PDMS are compressional bulk waves, which effectively heat the PDMS in a volumetric nature. The heating is rapid and uniform. We have found a range of SAW frequency where the acoustic absorption of PDMS becomes significant. The applicability of this fascinating phenomenon was assessed by developing a microheater specifically aimed at meeting the demand for a robust yet easy, inexpensive, portable, and disposable heating device for ‘lab-on-a-chip’ systems.

Lab-on-a-chip systems are broadly applicable to sophisticated biophysicochemical processes across a variety of science and engineering fields. Microfluidic thermal management in such systems has long been a major issue^6^. Microheaters have demonstrated crucial importance in diverse applications that include nucleic acid amplification^7^, cell lysis^8^, gas sensors^9^, temperature gradient focusing^10^,^11^, thermotaxis^11^, thermophoresis^12^,^13^, synthesis of micro and nanostructures^14^, and polymer synthesis^15^. Microheaters can be classified as a contact or a non-contact type according to whether the thermal elements are integrated into a system to enable conductive heat transfer. The most common contact-heating technique utilizes Joule-heated metal films patterned on glass substrates to locally conduct heat into fluid channels^7^. The microheaters are usually located inside the microchamber to minimize the temperature gradient, offering fast and accurate temperature control of the liquid samples. Yet the approach is optically non-transparent and prone to electrolysis in the liquid samples^16^. Moreover, the heating/cooling rates are potentially limited by the added thermal mass of the microheaters and the substrates, not by nanoliters of the fluid volume. Furthermore, the direct patterning of the heater on the chip makes the heater a part of the disposable component, increasing the cost and complexity of the disposable component for users. Here, the use of relatively expensive metals such as Pt and Au are usually required to minimize the chemical reaction between the metals and the liquid samples and increase the temperature measurement accuracy. On the other hand, non-contact heating methods based on infrared (IR)-mediated radiation^17^ or microwave dielectric heating^18^ enable noninvasive yet selective heating of a target fluid volume to produce faster thermal cycling^19^,^20^. As for IR-based heating, lens and filters can be employed to focus light and eliminate wavelengths (e.g., ultraviolet) that could heat other materials or chemically interfere with the liquid samples^21^. The devices equipped with an IR-source such as tungsten lamp are bulky and cannot be considered completely disposable or portable. Miniaturized IR lasers could be employed to overcome the limitations^21^. Microwave dielectric heating utilizes viscous heat dissipation from molecular motion within the liquid dielectric as the polar molecules will continuously rotate to align themselves in an oscillating electromagnetic field. Microwave directly heats the liquid, achieving fast heating ramp rate^22^. The power consumption was an order of magnitude lower than those reported for IR or Peltier heating^23^. A limitation of the microwave heating system is its complexity. Efforts are required to miniaturize the benchtop instruments needed for microwave heating such as microwave power source, microwave signal generator, and network analyzer to constitute a portable and disposable micro total analysis system^23^. The challenges may be overcome by adopting planar microwave circuitry from the advanced wireless technology and microelectronics^24^.

Here, we introduce a fast, accurate, conductive and transparent heating system. This approach employs a PDMS-based conventional surface acoustic wave microfluidic system and does not require additional complex processes to the fabrication. This powerful technique can even induce turbulence into a fluid sample, thereby enhancing mixing^25^ and achieving a uniform temperature distribution within the sample volume. The technique relies on heat dissipation from vibration damping of polydimethylsiloxane (PDMS) induced by piezo-actuated surface acoustic waves (SAWs). The heating system comprises PDMS microchannels bonded onto a LiNbO_3_ piezoelectric substrate. Regularly spaced interdigital transducers (IDTs) are patterned on the substrate through a single mask via a single-step lift-off lithography process. As SAW forms are determined by the designed patterns of IDTs, we can generate multiple waveforms, including pulsed or continuous, standing or traveling, focused or defocused, with different wavelengths and at multiple positions on a single chip, thereby synchronizing a variety of heat transfer operations at multiple positions (See Supplementary Video S1 and S2). The acoustic waves that propagate through a substrate’s surface readily couple with the PDMS, leak ultrasonic power into the PDMS in the form of longitudinal bulk waves called leaky SAWs, and produce heat through molecular oscillations (Fig. 1a).

## Results and Discussion

**Figure 1:**
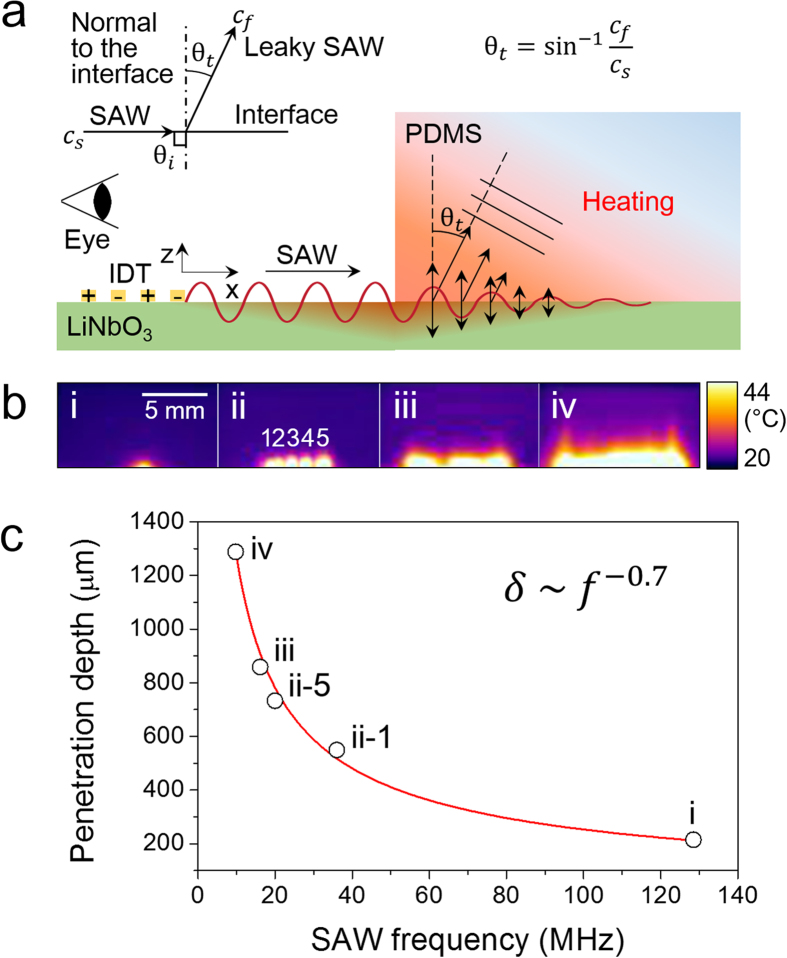
**a**, Schematic diagram showing that the IDT-driven surface acoustic waves refracted into the PDMS slab at an angle (*θ*_*t*_) (leaky Rayleigh wave) and induced heating. **b**, Infrared images showing the penetration depth at various SAW frequencies. The actuated frequencies and the type of IDT used were (i) 128.5 MHz (focused), (ii) (1) 36, (2) 32, (3) 28, (4) 24, and (5) 20 MHz (slanted; images stacked to a maximum intensity projection), (iii) 16.1 MHz (straight), and (iv) 9.8 MHz (straight), respectively. **c**, Plot showing the dependence of the penetration depth (defined here as the half-power depth) as a function of the SAW frequency.

We imaged the heating of PDMS caused by the penetration of the leaky SAWs (Fig. 1b). We placed a thick slab of PDMS on piezoelectric substrates with IDTs having different finger gap periods. Upon the actuation of the IDTs, an infrared camera with the view angle parallel to the substrate surface was used to record movies of the heating of the PDMS slab. The images in Fig. 1b were collected as snapshots from the movies upon the simultaneous satisfaction of two conditions: the maximum temperature reached 68 °C and exactly 1 sec had passed since the heating was initiated. The input power was adjusted to meet those conditions. As leaky SAWs propagate in the PDMS, they are attenuated to have a limited depth of penetration due to the viscosity-associated thermal dissipation of energy. In our experiment, the penetration depths were measured as the vertical length of the white region at which the temperature increase reached its half-maximum value (Fig. 1b). The penetration depths of the leaky SAWs were found to follow the power law^26^ (Fig. 1c),


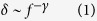


where *δ* is the penetration depth, *f* is the acoustic wave frequency, and *γ* is a real non-negative material parameter fitted to be approximately 0.7 from the experimental data (See Supplementary Table S1). This relation supports the model that PDMS heating originates from acoustic rather than electromagnetic absorption because the electromagnetic attenuation constant for a low-loss dielectric material is 1^27^. The penetration depths measured at frequencies from 9.8 MHz to 128.5 MHz ranged from 1290 μm to 210 μm, sufficient to cover most microchannel sizes. Heating was rapid (exceeding 2,000 K/s, Supplementary Video S3) and fairly uniform throughout the region.

**Figure 2:**
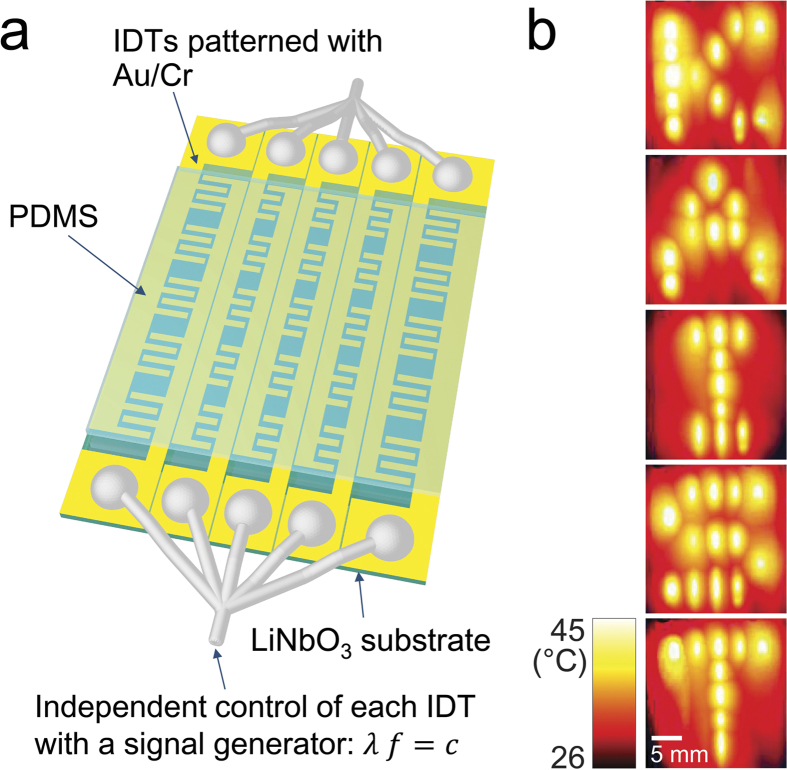
**a**, Schematic diagram showing the fabrication of the device that demonstrates pixel-by-pixel control over the temperature across a 2D plane. **b**, Infrared images showing the selective heating of each heat source pixel. Each pixel was sequentially heated to pattern the letters ‘K-A-I-S-T’, as shown in the stacked images of the maximum intensity projection.

Effective SAWs are produced only when the product of the IDT finger period (*λ*) and the electronic alternating current (AC) frequency (*f*) is equal to the speed of sound (*c*) in the LiNbO_3_ substrate, i.e., *λf* = *c*. The one-to-one correspondence between the IDT finger period and the driving AC frequency is extremely useful in that each IDT may be independently controlled to have its own finger period upon application of an AC having a matching frequency. Each IDT could function as an independently controlled heat source pixel. Moreover, multiple IDTs could be simultaneously actuated by time-sharing control by a single signal generator. Thus, a two-dimensional (2D) array of independently controllable small hot spots can be built by laying out a series of IDTs having all different finger periods. An example of this is illustrated in Fig. 2. A thin (~700 μm) slab of PDMS was placed on the LiNbO_3_ substrate on which 25 straight IDTs were patterned to have different finger gap periods each other. The IDTs are 2.2 mm in width by 4 mm in height in the 1^st^ and 5^th^ columns and 1.4 mm by 4 mm in the other columns. The finger gap periods of each IDT are 59 μm (top), 57 μm, 41 μm, 100 μm, 114 μm (bottom) for the 1^st^ column, 62 μm, 54 μm, 42 μm, 93 μm, 133 μm for the 2^nd^ column, 65 μm, 52 μm, 43 μm, 86 μm, 160 μm for the 3^rd^ column, 68 μm, 50 μm, 45 μm, 81 μm, 200 μm for the 4^th^ column, and 72 μm, 48 μm, 47 μm, 76 μm, 266 μm for the 5^th^ column. The IDTs in each column shares the same electrodes and the wires from those electrodes are tied together and connected to a signal generator. We controlled the temperature of each pixel independently, sequentially heating up only the pixels that pattern the letters ‘K-A-I-S-T’. All the pixels constituting the letters could not be heated at the same time enough to increase the temperature of PDMS by tens of Celsius because the power applied to the system was limited.

**Figure 3:**
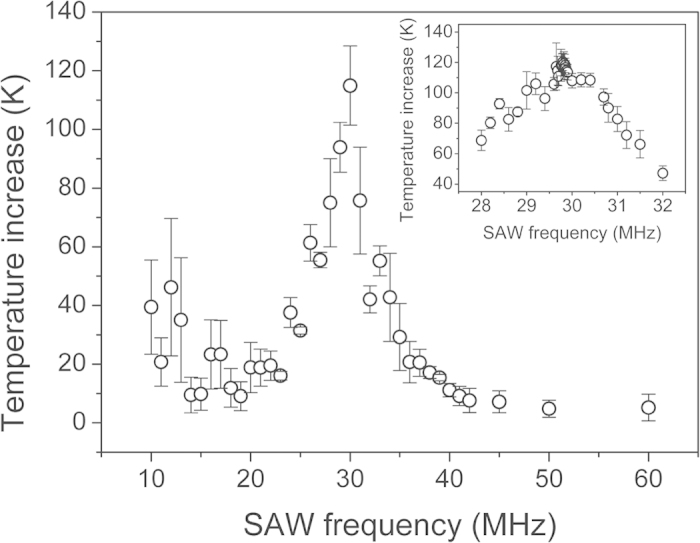
Plot showing the measurements of the PDMS temperature increase as a function of the SAW frequency over the range 10–60 MHz. The inset shows the temperature increase measurements at SAW frequencies from 28 to 32 MHz. The inset provides a finer profile of the loss factor around the peak. Slanted IDTs were used in the experiments. The finger gap periods were 66–800 μm in the main graph and 125–222 μm in the inset. The temperature increase measurements for the latter IDT to the full extent of the frequency are shown in Supplementary Figure S1. Error bars indicate the standard deviation from at least four independent measurements.

The heating is SAW frequency-dependent. Acoustothermal energy dissipation of the viscoelastic PDMS reaches a local maximum if the polymer relaxation period matches the period of the cyclic loading. The intrinsic properties of a material relate to the loss factor, which is defined as^28^:


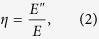


where *E* and *E* ˝ are the real and imaginary parts of the complex Young’s modulus, respectively. The loss factor represents the ratio of the energy lost per cycle to the peak potential energy. Therefore, the loss factor is proportional to the energy dissipated^28^ and thus, to the temperature increase in the material,


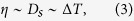


where *D*_*s*_ is the energy dissipated per cycle and Δ*T* is the temperature increase in the PDMS. As the loss factor depends on the frequency^28^, the profile of the loss factor can be obtained by measuring the temperature increase in the PDMS at different frequencies (Fig. 3). Figure 3 shows a sharp peak in the temperature increase at around 30 MHz. This peak indicated that the heating effects were rapid and energy-efficient if the signals applied to the IDTs were tuned to this frequency. A 30 MHz frequency is advantageous in that the IDT feature size is neither too big to fit onto a microchip nor too small, requiring thousands of dollars in costs for the high-resolution manufacture.

The functionality of the heater was assessed by performing a continuous flow polymerase chain reaction (CFPCR) for DNA amplification. The CFPCR provides a platform on which the PCR mixture is continuously pumped, rather than remaining stationary in a thermocycling chamber, so that it flows through different temperature zones to undergo denaturation, annealing, and extension, in order^29^. The continuous flow system is most suitable for lab-on-a-chip applications because the thermocycling is rapid, high-throughput, and can be easily integrated with upstream and downstream processes^20^. Significant limitations of the CFPCR devices have hindered its widespread use. The fixed layout of the channels and the heating system also fixes the number of PCR cycles and the relative time durations of each process^20^.

**Figure 4:**
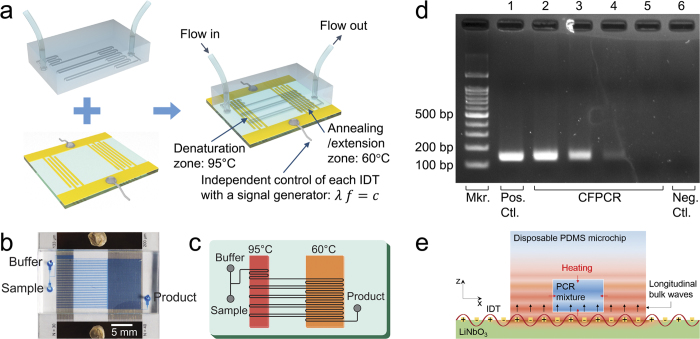
**a**, Schematic diagram showing the fabrication of a CFPCR chip. The PCR mixture flowing through the microchannels cycled between the denaturation zone (95 °C) and the annealing/extension zone (60 °C). The temperature of each zone was independently controlled by time-sharing two signals from a single signal generator. **b**, Photographic image of a CFPCR chip in which the microchannels are filled with blue ink (erioglaucine disodium salt, Sigma Aldrich, MO, USA). **c**, Schematic diagram describing the layout of the microchip for a two-step CFPCR. **d**, Fluorescent images of the gel electrophoresis results, obtained using a charge-coupled device camera (Gel Logic 200 Imaging System, Kodak, Seoul, Korea). The leftmost lane is a 100 bp DNA ladder. Lane 1: Reference PCR (positive control). Lane 2 to 5: CFPCR at increasing flow rates: 30, 60, 120, and 240 μL/hr, which corresponded to cycling times of 12, 6, 3, and 1.5 min, respectively, for 30 cycles. Lane 6: Negative control; CFPCR run using a template-free PCR mixture at a flow rate of 100 μL/hr (as in lane 3). **e**, Schematic diagram showing the conductive heat transfer from the PDMS microchip into the PCR mixture through the four sides of the microchannels.

A dynamic control of the heater layout for the CFPCR system could overcome the limitations. The heater can control pixel-by-pixel the temperature on a 2D plane. As the heating system permits adjustments to the area of each temperature zone, thereby controlling the relative time durations associated with each PCR process. One can activate or deactivate reactions in a microchannel by turning on/off the corresponding heat source pixels, thereby controlling the total number of PCR cycles. These features could be used to optimize the PCR protocols in a continuous flow system just as commercial thermal cyclers do. This optimization task is left for future work, as it requires a tremendous effort for the system development and optimization. Here, we demonstrate an ultrafast CFPCR system based on a rather simple chip design, as depicted in Fig. 4a,b. The details of the microfluidic CFPCR device fabrication are described in the Methods section. Two straight IDTs were fabricated on the substrate: one for denaturation (95 °C) and the other for annealing/extension (60 °C). The reaction mixture flowed back and forth between the two zones through the microchannels over 30 thermal cycles (Fig. 4c). The buffer was introduced between the sample runs to wash the microchannels, thereby preventing cross-contamination. The ratio of the denaturation and annealing/extension times in the two-step PCR was 1:4. The chip was run with flow rates ranging from 30 to 240 μL/hr, yielding a total flow-through time of from 12 to 1.5 min, for a fixed 30 cycles. The collected samples were analyzed by electrophoresis in a 2% agarose gel stained with RedSafe^TM^ (Intron Biotechnology, Seongnam, Korea) (Fig. 4d). The results demonstrated that effective PCR could be performed extremely rapidly (in less than 3 min) using the microheater, yielding products comparable to the size given by a commercial thermal cycler.

The PDMS microchip demonstrated here is truly disposable because the microchannels were closed using a thin layer of PDMS at the bottom and were brought into contact with the substrate by reversible bonding (Fig. 4e). The penetration depths for the two IDTs are calculated as about 600 μm and 800 μm respectively according to equation (1), which was enough to cover the microchannels. The sound waves that took the form of longitudinal bulk waves penetrated through the thin layer from the bottom of the chip and reached the ceiling and walls of the microchannels, thereby heating up the four sides of the microchannels. We performed an experiment to demonstrate the phenomenon. We showed that pieces of 50 μm-thick PDMS floated on top of PDMS/water layers with 400 μm thickness in total were heated by acoustic absorption, which implies that the leaky SAWs penetrated through the PDMS/water layers and reached the floating PDMS pieces (See Supplementary Figure S2 and Supplementary Video S4). The low thermal conductivity of PDMS enhanced the thermal efficiency. The power consumption was maintained below 6 W throughout the CFPCR experiments. The ultrafast CFPCR system can be made portable through the use of a palm-sized electronic driver circuit^30^ and battery-powered syringe pumps. This approach is applicable to heaters, actuators, or sensors for microelectromechanical systems, optofluidics, or paper microfluidics in the fields of food science, biochemical research, and medicine, where there is a need for heating capabilities beyond the limits of current heating technologies.

## Methods

### Microfluidic CFPCR device fabrication

The microfluidic CFPCR device was prepared using a microchannel-molded PDMS chip mounted on an IDT-patterned LiNbO_3_ substrate. The channel size for the CFPCR reactor was 50 μm in width, 71 μm in depth, and 1.69 m long, yielding a total reactor volume of 6.0 μL. The IDTs and electrodes were patterned by depositing layers of Au/Cr (1000/300 Å) through an e-beam evaporation lift-off process onto a 500 μm thick 128° y-x cut x-propagating LiNbO_3_ substrate (MTI Korea, Seoul, Korea)^31^. The electrodes were connected to wires using silver epoxy (EPO-TEK® H20E, Epoxy Technology, MA, USA). The PDMS microchip was fabricated by standard soft photolithography techniques using SU-8 replica molding protocols. The PDMS base (Sylgard 184A, Dow Corning, MI, USA) and curing agent (Sylgard 184B, Dow Corning, MI, USA) were used in a 10:1 ratio, poured over the SU-8 mold, and cured at 90 °C for over 2 hr. The thin PDMS layer, which was bonded to the microchip to close up the microchannels, was prepared using the same PDMS mixture and a silicon wafer silanized with trichloro(1H, 1H, 2H, 2H-perfluorooctyl)silane (Aldrich, MO, USA). The wafer was spin-coated (500 rpm, 30 sec) with the mixture to form a 200 μm-thick layer, cured at 90 °C for over 2 hr, and brought into contact with the bottom surface of the microchip after oxygen plasma treatment. The microchip was then cut from the wafer and positioned on top of the LiNbO_3_ substrate. The silanization of the wafer permitted separation of the cured PDMS layer from the wafer surface. Adhesive tapes (Scotch® Magic^TM^ Tape, 3M Korea, Seoul, Korea) were used to tightly bind the PDMS microchip and the substrate together because the microchip tended to swell due to heat expansion during the PCR experiments and detach from the substrate.

### Device operation

The microheaters were actuated using high-frequency (~MHz) AC signals produced by an RF signal generator (N5181A, Agilent Technologies, CA, USA) and amplified by a power amplifier (LZY-22+, Mini-Circuits, NY, USA). The amplified signals comprised one or multiple AC components, each with its own frequency, amplitude, and duration. MATLAB codes were used to produce such complex time-shared continuous signals and to manipulate these signals based on feedback from the temperature read-out measurements. The temperature distribution in the microsystem was monitored and measured using an infrared camera (T640, FLIR Systems, OR, USA). The emissivity on the camera was set to 0.95 both for an adhesive tape surface and PDMS surface. The temperatures required to prepare the denaturation, annealing, and extension zones were maintained with a temporal resolution of ±1 °C. The sample fluids were injected using a syringe pump (neMESYS, Cetoni GmbH, Korbussen, Germany) through the inlet ports and were collected from the outlet port.

### CFPCR

The DNA template examined here was a 48,502 bp *λ*-DNA c1857 Sam7 template (Bioneer, Daejeon, Korea). Primers were designed to produce a 134 bp amplicon from the target^32^ (Bioneer, Daejeon, Korea). The sequences of the forward and reverse primer were 5′-TGA TTC TGT TCC GCA TAA TTA CTC C-3′ and 5′-CAC CAA TGC TGA GAT AGC TGA AG-3′, respectively. The PCR mixture (300 μL) contained 150 μL Power SyBR® Green PCR Master Mix (Life Technologies Korea, Seoul, Korea), 135.8 μL ultrapure water, 10 μL 30 μg/μL bovine serum albumin (BSA; Life Technologies Korea, Seoul, Korea), 1.5 μL 100 μM forward primer, 1.5 μL 100 μM reverse primer, and 1.2 μL 500 ng/μL DNA template. BSA was used for a dynamic coating to chemically modify the microchannel walls and prevent the adsorption of polymerases onto the walls. Prior to performing the CFPCR experiments, a 10 μg/μL BSA solution was run through the microchannels over 1 hr to coat the walls. The buffer solution (GeneAmp® PCR Buffer II, Life Technologies Korea, Seoul, Korea) was run through the system between sample runs to prevent cross-contamination. After each run, the size and yield of the collected PCR products were estimated after gel electrophoresis by comparison with a 100 bp DNA marker (ELPIS Biotech, Daejeon, Korea). As a reference, the PCR mixture was run on a fast commercial thermal cycler (GeneAmp® PCR system 9700, Applied Biosystems, Seoul, Korea) with an overall 50 min run-time for 30 cycles.

## Additional Information

**How to cite this article**: Ha, B. H. *et al*. Acoustothermal heating of polydimethylsiloxane microfluidic system. *Sci. Rep*. **5**, 11851; doi: 10.1038/srep11851 (2015).

## Supplementary Material

Supplementary Information

Supplementary Video S1

Supplementary Video S2

Supplementary Video S3

Supplementary Video S4
