# Supplementary material for: Acoustothermal heating of polydimethylsiloxane microfluidic system
Source: Sci Rep. 2015 Jul 3;5:11851. doi: 10.1038/srep11851 (PMC4490350; doi:10.1038/srep11851)
Supplement: Supplementary Information [file srep11851-s1.pdf]

Acoustothermal heating of polydimethylsiloxane microfluidic system

Byung Hang Ha[1], Kang Soo Lee[1], Ghulam Destgeer[1], Jinsoo Park[1], Jin Seung Choung[1], Jin Ho Jung[1], Jennifer Hyunjong Shin[1] & Hyung Jin Sung[1]*

[1]Department of Mechanical Engineering, KAIST, 291 Daehak-ro, Yuseong-gu, Daejeon 305-701, Korea

Supplementary information

Supplementary Figure S1

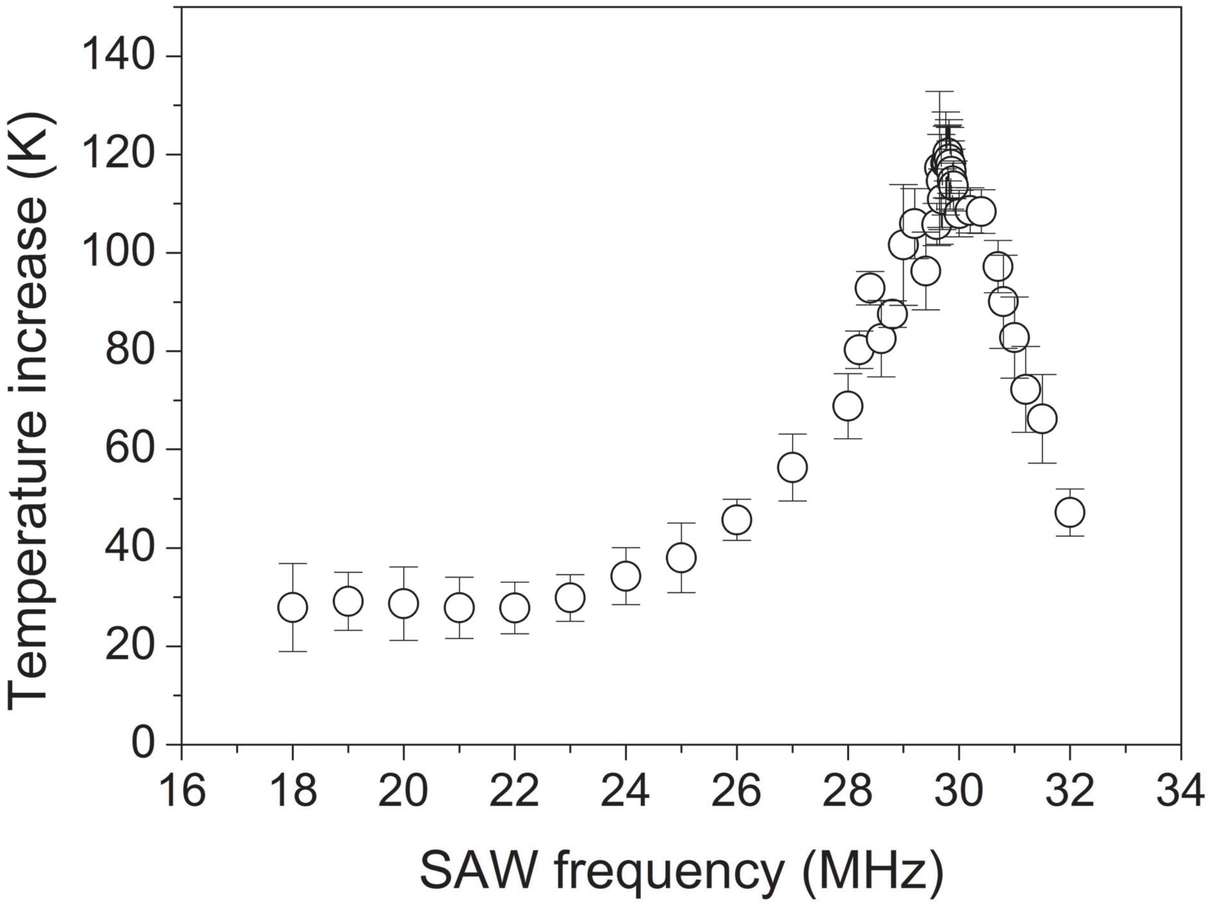

Title: Loss factor profile as a function of SAW frequency over the range 18–32 MHz

Plot showing the measurements of the temperature increase in the PDMS as a function of the SAW frequency over the range 18–32 MHz. Slanted IDTs with 125–222 μm finger periods were used in the experiments. The results confirmed the existence of a peak in the loss factor profile at around 30 MHz. Error bars indicate the standard deviations from at least four independent measurements.

Supplementary Figure S2

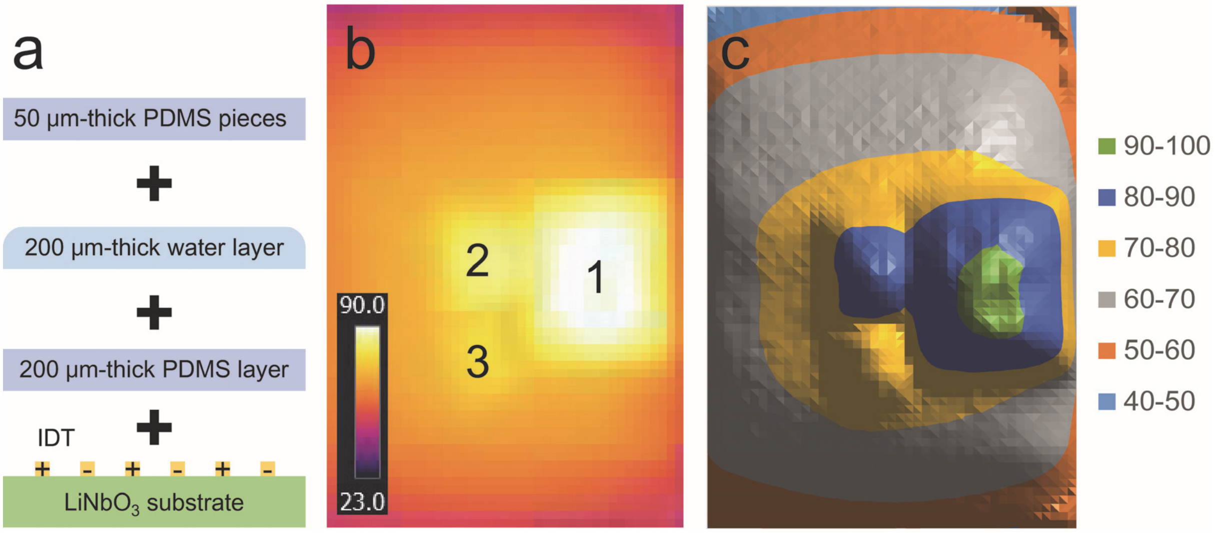

Title: Demonstration of the leaky SAW penetration into a ceiling of microchannels

a, Schematic diagram showing the fabrication of an experimental setup. First, a 4 mm-wide, 6 mm-long and 200 µm-thick PDMS layer was prepared and surface-treated by oxygen plasma. Second, the PDMS layer was placed on top of an IDT having 133 µm finger gap periods. Third, 4.8 µl of pure water was spread over the PDMS layer, forming a water layer with the thickness of about 200 µm. Fourth, three pieces of 50 µm-thick PDMS were floated on the water surface. **b**, Infrared image demonstrating that the leaky SAWs generated from the IDT penetrated through the layers of PDMS and water, reached the floating PDMS pieces, and heated them up. **c**, Temperature contour graph for the infrared measurement showing that the temperature of the floating PDMS pieces were much higher than that of the water, which demonstrates that the floating PDMS pieces were directly heated up by the SAWs.

Supplementary Video S1

Title: Heating of polydimethylsiloxane (PDMS) by multiple waveforms from focused IDTs

Heating of PDMS was observed by an infrared camera (A325sc, FLIR Systems, OR, USA). We can generate multiple focused waveforms, including pulsed or continuous, standing or traveling, synchronizing multi-heat transfer at multiple positions on a single chip. Heating of a piece of PDMS by three types of SAWs is demonstrated in the video. First, continuous traveling SAW generated from the focused IDT on the right heats the PDMS. Second, continuous standing SAWs generated from the focused IDTs at the both sides heat the PDMS. Strictly speaking, the waves would be absorbed by the PDMS and would not form a standing wave in the experiment. However, the waves would form a standing wave if the PDMS piece were as narrow as an order of the SAW wavelengths. Third, pulsed standing SAWs generated from the focused IDTs at the both sides heat the PDMS. The period is 2 sec.

Supplementary Video S2

Title: Heating of polydimethylsiloxane (PDMS) by multiple waveforms from a slanted IDT

Heating of PDMS was observed by an infrared camera (A325sc, FLIR Systems, OR, USA). We can generate multiple defocused waveforms with different wavelengths at multiple positions on a single chip, synchronizing multi-heat transfer at multiple positions. Heating of a piece of PDMS by five SAWs is demonstrated in the video. Five positions on the slanted IDT (finger gap periods ranging from 125 μm to 222 μm) are actuated by the application of time-shared five signals with five different frequencies (18, 20, 22.5, 25.5, and 30 MHz, respectively) from a single signal generator. All the signals were given with the same input power, 4 W.

Supplementary Video S3

Title: Heating rate measurement for polydimethylsiloxane (PDMS)

A heating rate of PDMS measured to be over 2,260 K/s by an infrared camera (T640, FLIR Systems, OR, USA). As soon as surface acoustic waves were generated from the LiNbO3 substrate, they coupled with the PDMS slab reversibly bonded on top of the substrate and rapidly heated it up. The 30 frames per second infrared camera measured the temperature rise of 74.6 K for 33 ms, giving the heating rate of 2,260 K/s. However, the heating rate would actually be higher than that because the camera was not able to measure temperature over 160°C and the measurement of the rising temperature was topped off at 160°C.

Supplementary Video S4

Title: Demonstration of the leaky SAW penetration into a ceiling of microchannels

Heating of PDMS pieces floating on top of a water layer by acoustic absorption was observed through infrared imaging (A325sc, FLIR Systems, OR, USA). The floating 50 μm-thick PDMS pieces and the 200 μm-thick PDMS layer beneath the water layer represent a ceiling and a bottom of microchannels, respectively. The video demonstrates that the leaky SAWs originated from the IDT penetrated through the layers of PDMS and water, reached, and heated the floating PDMS. The movement of the PDMS pieces were observed due to the presence of the leaky SAWs in the water. This implies that the SAW-driven heating of liquid in a closed microchannel is done by conduction through the four walls of the microchannel.

Supplementary Table S1

Title: Penetration depths measurement data

The penetration depths were measured by infrared imaging (T640, FLIR Systems, OR, USA). Snapshot images were taken when the maximum temperature reached 68°C and exactly 1 sec had passed since the heating was initiated. The data indicate the vertical length of the area where the temperature increase reached its half-maximum value. Repeated experiments with different setups produced the same results.

SAW frequency (MHz)	Penetration depth (μm)
9.8000	1290
16.132	860
20.000	730
36.000	550
128.50	210
